# Age, sex and disease-specific associations between resting heart rate and cardiovascular mortality in the UK BIOBANK

**DOI:** 10.1371/journal.pone.0233898

**Published:** 2020-05-29

**Authors:** Zahra Raisi-Estabragh, Jackie Cooper, Rebekah Judge, Mohammed Y. Khanji, Patricia B. Munroe, Cyrus Cooper, Nicholas C. Harvey, Steffen E. Petersen

**Affiliations:** 1 William Harvey Research Institute, NIHR Barts Biomedical Research Centre, Queen Mary University of London, London, United Kingdom; 2 Barts Heart Centre, St Bartholomew’s Hospital, Barts Health NHS Trust, London, United Kingdom; 3 Imperial College Healthcare Trust, St Mary’s Hospital, London, United Kingdom; 4 MRC Lifecourse Epidemiology Unit (MRCLEU), Southampton, United Kingdom; 5 NIHR Southampton Biomedical Research Centre, University of Southampton and University Hospital Southampton NHS Foundation Trust, Southampton, United Kingdom; 6 NIHR Oxford Biomedical Research Centre, University of Oxford, Oxford, United Kingdom; Shanghai Institute of Hypertension, CHINA

## Abstract

**Objective:**

To define the sex, age, and disease-specific associations of resting heart rate (RHR) with cardiovascular and mortality outcomes in 502,534 individuals from the UK Biobank over 7–12 years of prospective follow-up.

**Methods:**

The main outcomes were all-cause, cardiovascular, and ischaemic heart disease mortality. Additional outcomes included incident acute myocardial infarction (AMI), fatal AMI, and cancer mortality. We considered a wide range of confounders and the effects of competing hazards. Results are reported as hazard ratios (HR) for all-cause mortality and sub-distribution hazard ratios (SHR) for other outcomes with corresponding 95% confidence intervals (CI) per 10bpm increment of RHR.

**Results:**

In men, for every 10bpm increase of RHR there was 22% (HR 1.22, CI 1.20 to 1.24, p = 3×10^−123^) greater hazard of all-cause and 17% (SHR 1.17, CI 1.13 to 1.21, p = 5.6×10^−18^) greater hazard of cardiovascular mortality; for women, corresponding figures were 19% (HR 1.19, CI 1.16 to 1.22, p = 8.9×10^−45^) and 14% (SHR 1.14, CI 1.07 to 1.22, p = 0.00008). Associations between RHR and ischaemic outcomes were of greater magnitude amongst men than women, but with similar magnitude of association for non-cardiovascular cancer mortality [men (SHR 1.18, CI 1.15–1.21, p = 5.2×10^−46^); women 15% (SHR 1.15, CI 1.11–1.18, p = 3.1×10^−18^)]. Associations with all-cause, incident AMI, and cancer mortality were of greater magnitude at younger than older ages.

**Conclusions:**

RHR is an independent predictor of mortality, with variation by sex, age, and disease. Ischaemic disease appeared a more important driver of this relationship in men, and associations were more pronounced at younger ages.

## Introduction

Cardiovascular disease (CVD) is the most common cause of ill-health and death in the world [1]. Understanding novel disease markers is key to improved disease prevention, treatment, and prognostication. A positive association between resting heart rate (RHR) and mortality has been repeatedly demonstrated in the literature. This association has been replicated in diverse study populations, including cohorts with known CVD, and in healthy populations [2–6]. There is general agreement, that in men, this relationship is driven by excess CVD mortality. However, reports for women are conflicting, varying from a more pronounced effect [7], to a weakened or absent association [8–11]. Despite wide recognition of differential patterns of CVD in men and women [12], differential associations of RHR by sex have not been adequately studied. There is under-representation of women in existing reports, with several studies limited to men-only cohorts [13–15]. Similarly, there are inconsistent reports of the modifying effect of age on RHR-mortality associations [10,13,16–18]. Previous studies of potential sex and age interactions have been limited by participant numbers, lack of consideration of specific causes of death, and an inability to adequately control for covariates and competing outcomes.

RHR has potential as an inexpensive and reliable risk predictor. We therefore sought to define the sex, age, and cause-specific associations of RHR with important cardiovascular and mortality outcomes in 502,534 participants from the UK Biobank (UKB) over 7–12 years of prospective follow-up. The main outcomes were all-cause, cardiovascular, and ischaemic heart disease (IHD) mortality. Additional outcomes included incident acute myocardial infarction (AMI), fatal AMI, and cancer mortality. The dataset permitted consideration of a wide range of potential confounders and the effects of competing hazards such as death or alternative causes of death.

## Methods

### Setting, participants, and recruitment: The UK Biobank

UKB is a large population-based research cohort, incorporating data from over 500,000 participants recruited aged 40–69 years between 2006–2010 from across the UK. Recruitment was through postal invite. Participants who were unable to provide consent or partake in data collection due to discomfort or illness were not enrolled. The UKB cohort and process are described in dedicated publications [19,20].

### Ethics

This study was covered by the ethical approval for UKB studies from the National Health Service (NHS) National Research Ethics Service on 17th June 2011 (Ref 11/NW/0382) and extended 10^th^ May 2016 (Ref 16/NW/0274).

### Baseline assessment and defining participant characteristics

All UKB participants completed a comprehensive baseline assessment of health and lifestyle by self-report questionnaire and nurse-led interview. Body mass index (BMI), smoking status, and level of physical activity were defined for the whole cohort. Smoking status was defined as a binary variable: current vs non-smokers. We derived a continuous value for the amount of physical activity measured in metabolic equivalent (MET) minutes/week calculated by weighting different types of activity by its energy requirements using values derived from the International Physical Activity Questionnaire (IPAQ) study [21]. We defined hypercholesterolaemia by self-report of a diagnosis of ‘high cholesterol’ or use of ‘cholesterol lowering medication’. Diabetes status was determined by participants' response to the binary questionnaire item ‘diabetes diagnosed by a doctor’. Hypertension was defined as self-reported regular use of ‘blood pressure medication’ or code of ‘essential hypertension’ assigned at the verbal interview. Additionally, we separately consider systolic blood pressure measured at baseline. We also considered self-reported use of heart rate modifying medications: beta-blockers, oral nitrates, non-dihydropyridine calcium channel blockers, amiodarone, digoxin, and flecainide (S1 Table).

### Measurement of RHR

RHR was measured according to a pre-defined standard operating procedure at the baseline UKB assessment visit, detailed in a dedicated document [22]. Measurement was taken in a dedicated room using the Omron 705 IT electronic blood pressure monitor (OMRON Healthcare Europe B.V. Kruisweg 577 2132 NA Hoofddorp). The participant was asked to sit with their feet parallel to each other, toes pointing forward and soles of feet resting flat on the floor. Measurement was taken from the left arm; the right arm was used if measurement from the left was not practical (amputee, shunt, mastectomy, axillary clearance). Restrictive clothing was loosened or removed from the upper arm. Staff members were instructed to avoid engaging participants in conversation. The participant was asked to place their arm on the desktop and breathe in and out slowly in a relaxed fashion. The correct cuff size was selected after measurement of the mid-point circumference of the upper arm. The operator would start the measurement by pressing start on the monitor. The blood pressure and pulse rate measures generated by the machine auto-populated to the participants’ electronic record. After completing the first measurement, the rubber inflation tubing was disconnected from the Omron monitor, the cuff was left in place but allowed to deflate completely. The participant was then asked to gently shake their arm, and open and close their hand a few times. A second reading was obtained after waiting for at least 1 minute. A timer in the computer ensured that the second pressure reading could not be taken before 1 minute had elapsed. We used the mean of these two heart rate readings in the analysis.

### Ascertainment of outcomes

Linkages have been established with electronic Hospital Episode Statistics (HES) and death registers for the whole UKB cohort allowing prospective tracking of verified outcomes from baseline with conditions defined in accordance with the International Classification of Diseases (ICD). Further, UKB has produced algorithms to reliably identify incidence of selected illnesses through consideration of HES and death register data. UKB adjudication of “algorithmically defined” outcomes is detailed in a dedicated document [23].

We considered outcomes occurring from point of recruitment (2006–2010) to the latest UKB censor dates (mortality outcomes: 31/01/2018, incident AMI: 31/03/2017) giving follow-up duration of 7–12 years. We defined three main outcomes: all-cause mortality, CVD mortality, and IHD mortality. Secondary outcomes included: incident AMI, fatal AMI, and cancer mortality.

Mortality outcomes were defined from death register records of primary cause of death. CVD mortality was defined as primary cause of death attributed to CVD, which encompassed all ischaemic and non-ischaemic CVD. IHD mortality included all deaths primarily attributed to IHD, which includes fatal AMI as well as other complications such as ischaemic cardiomyopathy. Fatal AMI includes cases with AMI as the primary cause of death. Incident AMI was derived from algorithmically defined outcomes, AMIs occurring after the baseline UKB visit were considered. We include cancer mortality as a secondary outcome as an important representative of non-cardiac mortality, defined as primary cause of death attributed to any cancer.

### Statistical analysis

Statistical analysis was performed using R studio version 3.6.0 [https://www.R-project.org/] and Stata version 14 [StataCorp. 2015. *Stata Statistical Software*: *Release 14*. College Station, TX: StataCorp LP]. Models for all-cause mortality were fitted using Cox proportional hazards models. Other end-points (CVD mortality, IHD mortality, Cancer mortality, incident AMI, fatal AMI) were analysed using competing risks regression with proportional sub-distribution hazards fitted as per Fine and Gray competing risks regression model [24]. RHR was analysed as a continuous variable with mean RHR calculated in those with and without the event. Results for incident data are reported as hazard ratios (HR) for all-cause mortality, and sub-distribution hazard ratios (SHR) for all other endpoints, with corresponding 95% confidence intervals (CI) per 10bpm (beats per minute) increase in RHR. We tested for non-linearity of RHR effect by comparison of linear and non-linear (restricted cubic spline model with five knots at each quintile) models for all outcomes. When non-linearity was detected, we further characterised the relationship using restricted cubic spline models.

**Fig 1:**
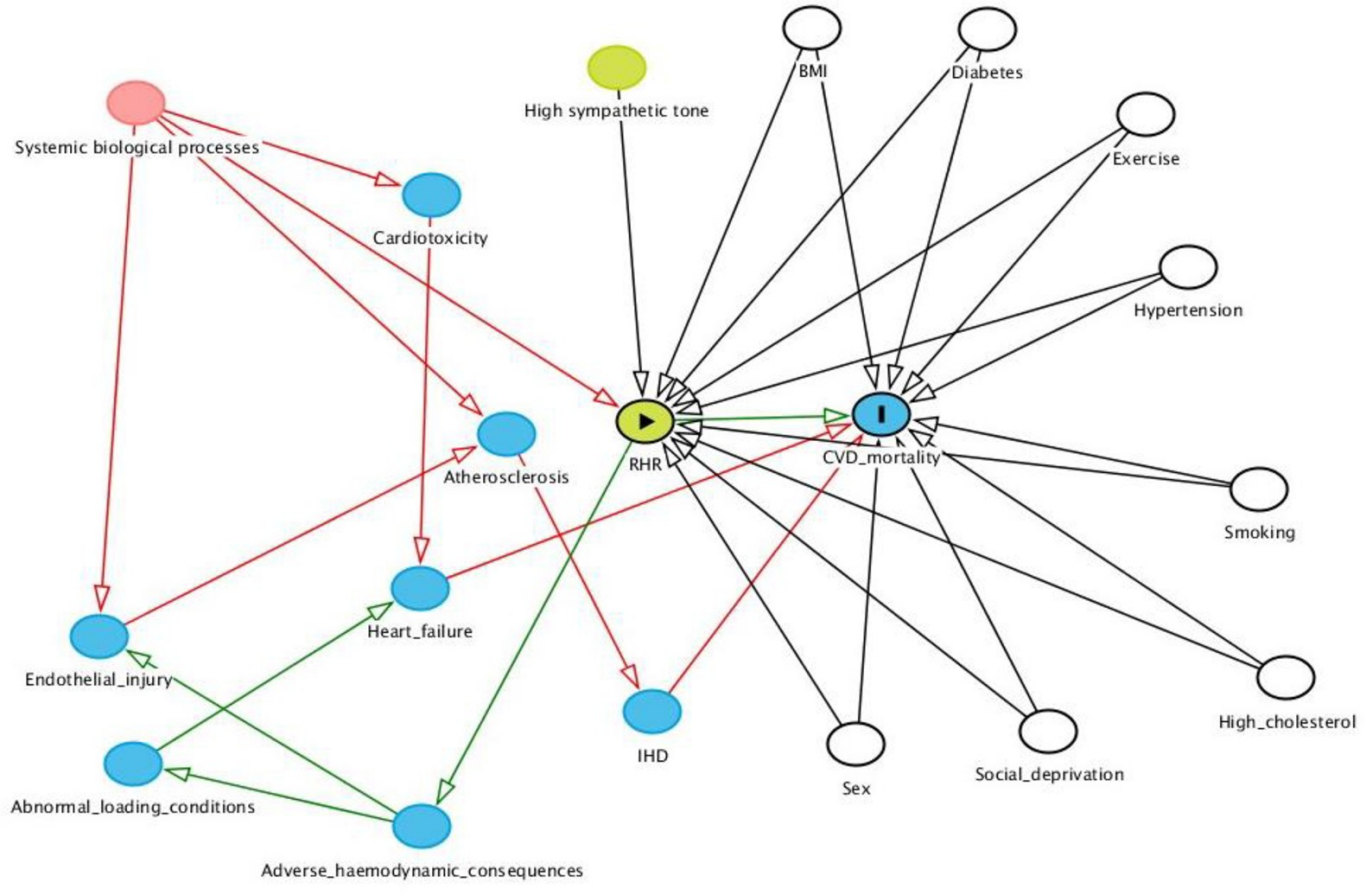
Directed acyclic graph of the potential relationships between cardiovascular disease and resting heart rate. BMI: Body mass index; CVD: Cardiovascular disease; IHD: Ischaemic heart disease; RHR: Resting heart rate. In this model: RHR = exposure and CVD mortality = outcome; Unshaded circles represent true confounders, all controlled for in fully adjusted model.

Covariates were selected based on existing literature, which we confirmed through preliminary analyses. We systematically considered potential causal pathways guided by existing knowledge of disease mechanisms (Fig 1). The following covariates are included in the fully adjusted models: age, sex, BMI, exercise, smoking, diabetes, hypertension, hypercholesterolaemia, social deprivation, and heart rate modifying medications. In a separate analysis, we present the models with systolic blood pressure reading in place of clinically diagnosed hypertension. There was no evidence of multicolinearity for any of the covariates (Variance Inflation Factor <2 for all variables).

The proportional hazards assumption was tested using the Schoenfeld residuals. Interactions with sex and age were investigated by adding interaction terms to the main model. Age stratified analysis is presented for all outcomes. The accepted level of statistical significance for non-interaction regressions, after correction for multiple comparisons using the Bonferroni correction, was set at p = 0.0008.

## Results

### Baseline characteristics of the population

RHR data was missing for 1,203 (0.2%) individuals, resulting in an analysis dataset comprising 228,594 men and 272,737 women. The women had median age of 57 (50–63) years and mean baseline RHR of 70.3 (10.6) bpm. The median age of the men was 58 (50–64) with a mean baseline RHR of 68.4 (11.9) bpm. As expected, there was a higher prevalence of cardiovascular risk factors in men compared to women with higher levels of smoking (13% vs 9%), diabetes (7% vs 4%), hypertension (33% vs 24%), and elevated cholesterol (24% vs 14%). Detailed baseline characteristics are presented in Table 1. We also present these baseline characteristics in quartiles of RHR for men and women (S2 Table).

**Table 1:** Baseline participant characteristics.

	Whole cohort *n* = 502,534	Men *n* = 228,594	Women *n* = 272,737
Age (years)	56.5(8.1)	56.7 (8.2)	56.3 (8.0)
Townsend deprivation index	-1.29 (3.10)	-1.25 (3.16)	-1.33 (3.04)
Current smoker	52979 (10.6%)	28612 (12.5%)	24367 (8.9%)
Body mass index (kg/m^2^)	27.4 (4.8)	27.8 (4.3)	27.1 (5.2)
Systolic blood pressure (mmHg)	137.9 (18.7)	140.9 (17.5)	135.3 (19.2)
Diastolic blood pressure (mmHg)	82.3 (10.1)	84.1 (10.0)	80.7 (10.0)
Diabetes	26,833 (5.3%)	16,271 (7.1%)	10,562 (3.9%)
Hypertension	141,019 (28.1%)	74,803 (32.6%)	66,216 (24.2%)
Hypercholesterolaemia	93,822 (18.7%)	55,722 (24.3%)	38,100 (13.9%)
Resting heart rate (bpm)	69.4 (11.3)	68.4 (11.9)	70.3 (10.6)
Physical Activity (metabolic equivalent minutes/week)	2043.5 (2239.5) 1370 [648 to 2586]	2191.6 (2490.4) 1413 [658 to 2772]	1907.0 (1970.5) 1308 [636 to 2445]
Follow-up (days):			
Mortality	9.0 [8.2–9.7]	8.9 [8.2–9.7]	9.0 [8.3–9.7]
Incident AMI	8.1 [7.5–8.8]	8.1 [7.4–8.8]	8.2 [7.5–8.8]

*Results are mean (standard deviation), number (percentage), or median [interquartile range]. AMI: acute myocardial infarction; bpm: beats per minute

### Follow-up duration and number of outcomes

Median duration of follow-up for mortality from baseline was 9.0 [8.2–9.7] years for mortality outcomes and 8.1 [7.5–8.8] years for incident AMI. Over this time period we observed 20,126 deaths and 8,605 cases of incident AMI. Full breakdown of the number of observed outcomes in men and women is presented in Table 2. Results of RHR-outcome association models are presented in Table 3. Models with systolic blood pressure as covariate in place of hypertension show almost identical results (S3 Table).

**Table 2:** Observed outcomes for the whole cohort, and for men and women separately.

	Whole cohort (*n* = 501,331)	Men (*n* = 228,594)	Women (*n* = 272,737)
All-cause mortality	20,126 (4.0%)	12,137 (5.3%)	7,989 (2.9%)
CVD mortality	4,197 (0.8%)	3,069 (1.3%)	1,128 (0.4%)
IHD mortality	2,285 (0.5%)	1,877 (0.8%)	408 (0.1%)
Fatal AMI	920 (0.2%)	741 (0.3%)	179 (0.1%)
Incident AMI	8,605 (1.7%)	6,069 (2.7%)	2,536 (0.9%)
Cancer mortality	11,167 (2.2%)	6,041 (2.6%)	5,126 (1.9%)
Follow-up (days): median [IQR]			
Death	9.0 [8.2–9.7]	8.9 [8.2–9.7]	9.0 [8.3–9.7]
AMI	8.1 [7.5–8.8]	8.1 [7.4–8.8]	8.2 [7.5–8.8]

*Results are presented as number of outcomes (percentage). AMI: acute myocardial infarction; CVD: cardiovascular disease; IHD: ischaemic heart disease; IQR: interquartile range.

**Table 3:** Cox proportional hazard models and sub-distribution hazard models for all RHR-outcome relationships^†^.

	Model 1: Univariate	Model 2: Age	Model 3: Age, smoking, exercise, BMI	Model 4: Model 3 + CVD risk factors^*^	Model 5: Model 4 + rate modifying medications^**^
All-cause mortality					
Men	1.25 (1.24 to 1.27)	1.26 (1.24 to 1.28)	1.21 (1.19 to 1.23)	1.19 (1.17 to 1.21)	1.22 (1.20 to 1.24)
p-value	2.5 ×10^−228^	6.7 ×10^−246^	5.2 ×10^−122^	7.9 ×10^−95^	3.0 ×10^−123^
Women	1.25 (1.23 to 1.28)	1.22 (1.20 to 1.25)	1.16 (1.14 to 1.19)	1.15 (1.13 to 1.18)	1.19 (1.16 to 1.22)
p-value	7.2 ×10^−119^	1.2 ×10^−98^	2.6 ×10^−35^	4.4 ×10^−32^	8.9 ×10^−45^
CVD mortality					
Men	1.21 (1.18 to 1.25)	1.22 (1.18 to 1.25)	1.14 (1.10 to 1.18)	1.10 (1.06 to 1.14)	1.17 (1.13 to 1.21)
p-value	4.6 ×10^−37^	4.0 ×10^−41^	1.9 ×10^−12^	6.0 ×10^−8^	5.6 ×10^−18^
Women	1.22 (1.16 to 1.29)	1.19 (1.13 to 1.26)	1.08 (1.01 to 1.17)	1.07 (1.00 to 1.15)	1.14 (1.07 to 1.22)
p-value	5.6 ×10^−13^	9.2 ×10^−11^	0.028	0.042	0.00008
IHD mortality					
Men	1.18 (1.14 to 1.23)	1.19 (1.14 to 1.23)	1.10 (1.05 to 1.15)	1.06 (1.01 to 1.11)	1.14 (1.09 to 1.19)
p-value	4.0 ×10^−17^	5.1 ×10^−19^	0.0001	0.015	1.2 ×10^−8^
Women	1.19 (1.08 to 1.30)	1.16 (1.06 to 1.27)	1.02 (0.87 to 1.12)	0.98 (0.87 to 1.10)	1.06 (0.94 to 1.18)
p-value	0.0004	0.002	0.82	0.67	0.36
Fatal AMI					
Men	1.16 (1.09 to 1.23)	1.17 (1.10 to 1.24)	1.08(1.00 to 1.16)	1.05 (0.98 to 1.12)	1.12 (1.05 to 1.21)
p-value	1.4 ×10^−6^	2.7 ×10^−7^	0.044	0.21	0.002
Women	1.05 (0.90 to 1.22)	1.03 (0.97 to 1.33)	0.85 (0.70 to 1.04)	0.85 (0.70 to 1.03)	0.92 (0.77 to 1.10)
p-value	0.52	0.70	0.11	0.09	0.36
Incident AMI					
Men	1.09 (1.07 to 1.11)	1.09 (1.07 to 1.11)	1.04 (1.01 to 1.06)	1.01 (0.99 to 1.04)	1.05 (1.02 to 1.07)
p-value	1.1 ×10^−15^	6.7 ×10^−16^	0.007	0.36	0.0003
Women	1.08 (1.04 to 1.13)	1.06 (1.02. to 1.11)	1.00 (0.95 to 1.05)	0.99 (0.94 to 1.04)	1.03 (0.98 to 1.08)
p-value	0.0001	0.002	0.89	0.72	0.28
Cancer mortality					
Men	1.20 (1.18 to 1.23)	1.21 (1.18 to 1.23)	1.18 (1.16 to 1.21)	1.17 (1.14 to 1.20)	1.18 (1.15 to 1.21)
p-value	9.9 ×10^−75^	7.9 ×10^−83^	1.6 ×10^−48^	1.8 ×10^−42^	5.2 ×10^−46^
Women	1.20 (1.17 to 1.23)	1.17 (1.15 to 1.20)	1.14 (1.11 to 1.18)	1.14 (1.10 to 1.17)	1.15 (1.11 to 1.18)
p-value	6.8 ×10^−48^	6.4 ×10^−39^	2.9 ×10^−17^	1.7 ×10^−16^	3.1 ×10^−18^

^**†**^Results are hazard ratio (95% confidence interval) for all-cause mortality and sub-distribution hazard ratio (95% confidence interval) for all other outcomes per 10 beat per minute increase in resting heart rate. Significance level is p-value <0.0008. Those with prevalent MI have been excluded from analysis of incident AMI and fatal AMI.

* CVD risk factors include: diabetes, hypertension, hypercholesterolaemia, Townsend deprivation index.

**rate modifying medications include: betablockers, non-dihydropyridine calcium channel blockers, oral nitrates, digoxin, flecainide, amiodarone. AMI: acute myocardial infarction; BMI: body mass index; CVD: cardiovascular disease; IHD: ischaemic heart disease

### Test for non-linearity of RHR effect

There was non-linearity in the effect of RHR on incident AMI in women (non-linear vs linear model: p-value = 0.002). There was no evidence of non-linearity in RHR effect for any the other outcome, or for incident AMI in men (S4 Table).

### Association between RHR and all-cause mortality

RHR was positively associated with all-cause mortality in both men and women (Table 3). In the fully adjusted model, a 10bpm increase of RHR was associated with 22% (HR 1.22, CI 1.20–1.24, p = 3 ×10^−123^) greater hazard of all-cause mortality in men and 19% (HR 1.19, CI 1.16–1.22, p = 8.9 ×10^−45^) greater hazard in women.

### Associations between RHR and cardiovascular outcomes

In both men and women, higher RHR was associated with increased hazard of CVD mortality. Every 10bpm increase of RHR was associated with 17% (SHR 1.17, CI 1.13–1.21, p-value 5.6 ×10^−18^) greater hazard of CVD mortality in men and 14% (SHR 1.14, CI 1.07–1.22, p-value 0.00008) greater hazard in women. Ischaemic aetiology was a significant driver of this relationship in men: 14% (SHR 1.14, CI 1.09–1.19, p-value 1.2 ×10^−8^) greater hazard of IHD mortality, 5% (SHR 1.05, CI 1.02–1.07, p-value 0.0003) greater hazard of incident AMI, and 12% (SHR 1.12, CI 1.05–1.21, p-value 0.002) greater hazard of fatal AMI per 10bpm increment of RHR (Table 3). However, for women, there was no significant association between RHR and IHD mortality or fatal AMI. There was evidence of non-linearity in the RHR effect on incident AMI in women (S4 Table) with restricted cubic spline models demonstrating a U-shaped association. We illustrated this relationship further by stratified analysis in quintiles of RHR (Table 4). For women, there was significantly increased hazard of incident AMI in the highest and lowest quintiles when compared to the middle quintile. There was no evidence of non-linearity of effect on incident AMI for men (linear vs non-linear model; p-value 0.52).

**Table 4:** The association of RHR with incident AMI in quintiles for men and women.

	HR (95% CI)*				
RHR (bpm)	<59	59–64	65–70	71–77	≥78
Men	0.96 (0.87–1.06)	0.97 (0.88–1.06)	1.00	1.06 (0.96–1.16)	1.15 (1.05–1.27)
	<62	62–67	68–72	73–78	≥79
Women	1.21 (1.02–1.42)	1.08 (0.91–1.28)	1.00	1.16 (0.98–1.36)	1.22 (1.04–1.43)

*HR as compared to middle quintile, fully adjusted model (age, smoking, BMI, exercise level, diabetes, hypertension, hypercholesterolaemia, smoking, BMI, Townsend deprivation index. rate modifying medications). AMI: acute myocardial infarction; bpm: beats per minute; CI: confidence interval; RHR: resting heart rate.

### Associations between RHR and cancer mortality

To compare an exemplar of important non-cardiac with cardiac drivers of all-cause mortality, we analysed the relationship between RHR and incident cancer mortality. In both men and women, higher RHR was associated with significantly greater hazard of cancer mortality (Table 3). A 10bpm higher RHR was associated with 18% (SHR 1.18, CI 1.15–1.21, p-value 5.2 ×10^−46^) greater hazard of cancer mortality in men and 15% (SHR 1.15, CI 1.11–1.18, p-value 3.1 ×10^−18^) greater hazard in women.

### Interaction with age

Tests for interaction were made with age as a continuous variable, but for ease of interpretation we present hazard ratios by age group. The age groups were chosen to create even deciles (<50years, 50-59years, >60years). We do not ascribe any clinical meaning to these age groups and would not advocate viewing these as thresholds. In age-stratified analyses (Table 5), there was a significant trend for greater effect size in younger participants for all-cause mortality [HR 1.30 (1.24–1.36) <50 years-old vs 1.15 (1.13–1.16) ≥60 years-old] and cancer mortality [HR 1.21 (1.13–1.30) <50 years-old vs 1.15 (1.13–1.18) ≥60 years-old]. The same age modifying effect was observed for incident AMI, with the association becoming null in the oldest age category [HR: 1.14 (1.06–1.22) <50 years-old vs 1.02 (0.99–1.05) ≥60 years-old].

**Table 5:** Age interactions and age stratified analyses for all outcomes.

Outcome	Age group (years)	*n*	HR (95% CI)*	Age RHR Interaction (p-value)**
All-cause mortality	<50	117,535	1.30 (1.24 to 1.36)	9.5 ×10^−15^
	50–59	166,778	1.26 (1.22 to 1.29)	
	≥60	217,018	1.15 (1.13 to 1.16)	
CVD mortality	<50	117,535	1.11 (0.99 to 1.24)	0.15
	50 to 59	166,778	1.28 (1.21 to 1.36)	
	≥60	217,018	1.15 (1.11 to 1.20)	
IHD mortality	<50	117,535	1.06 (0.91 to 1.24)	0.51
	50 to 59	166,778	1.24 (1.15 to 1.34)	
	≥60	217,018	1.11 (1.06 to 1.17)	
Incident AMI	<50	116,875	1.14 (1.06 to 1.22)	0.0004
	50 to 59	164,030	1.06 (1.02 to 1.10)	
	≥60	208,372	1.02 (0.99 to 1.05)	
Fatal AMI	<50	117,535	1.09 (0.84 to 1.40)	0.09
	50 to 59	166,778	1.26 (1.11 to 1.43)	
	≥60	217,018	1.05 (0.97 to 1.14)	
Cancer mortality	<50	117,535	1.21 (1.13 to 1.30)	0.0007
	50–59	166,778	1.22 (1.18 to 1.26)	
	≥60	217,018	1.15 (1.13 to 1.18)	

AMI: acute myocardial infarction; CVD: cardiovascular disease; IHD: ischaemic heart disease; RHR: resting heart rate.

*Results are Hazard ratio (95% Confidence interval) per 10bpm increase in heart rate.

## Discussion

### Summary of findings

In this large prospective study of over 500,000 individuals with 7–12 years follow-up, we confirm RHR as an independent predictor of all-cause and CVD mortality in both men and women. Ischaemic cardiac events appeared a consistent and significant driver of this relationship for men. For women, there was no significant relationship between RHR and fatal AMI or IHD mortality, and there was a non-linear effect on incident AMI. In both men and women, the excess mortality cannot be accounted for entirely by CVD events. We observed significant associations between RHR and increased cancer mortality. The effect of RHR was greater in younger individuals for all-cause mortality, cancer death, and incident AMI. Our findings add to the literature by demonstration of age, sex, and disease-specific aspects of the relationship in a large well-defined population.

### Comparison with existing studies

Elevated RHR has been repeatedly linked to increased all-cause mortality in multiple studies and in diverse populations [2–5]. However, the sex-differential pattern of this relationship is incompletely understood. Whilst existing literature is supportive of increased CVD mortality as the likely driver of the RHR-mortality association in men [13–15], reports in women are inconsistent [7–11]. Similarly, for men, ischaemic cardiac outcomes have been proposed as an important driver of mortality associations, whilst in women the evidence is conflicting [7–9]. In the largest reported cohort of women to date (*n* = 199,490), Tverdal et al. reported no association between RHR and risk of CVD or IHD mortality in women [11]. Conversely, a prospective cohort study of 129,135 women documented a positive association between RHR and ischaemic cardiac events, expressed as a composite of AMI and coronary death [25]. Several older studies with smaller sample sizes have reported increased hazard of CVD and IHD mortality in both men and women [9,18,26]. A pooled analysis of 12 cohort studies, suggests that for women, there is a positive association of RHR with CVD mortality, but not with ischaemic cardiac outcomes [17].

Taken as whole, our findings, in the largest cohort studied to date, confirm the contribution of ischaemic cardiac events to the RHR-mortality relationship in men, and suggest that non-ischaemic cardiac disease may be a more important mediator in women. Considering the point estimates and 95% CIs (Table 3), the possibility for a sex difference seems most convincing in relation to fatal AMI. For other ischaemic outcomes, this is less certain, as there is substantial overlap between the 95% CIs for these outcomes (IHD mortality, incident AMI) by sex. It was interesting to observe the U-shaped effect of RHR on incident AMI in women. This may be explained by sex differences in disease mechanisms; for instance, in women, coronary atherosclerosis may manifest as arrhythmic disease with consequently higher risks of incident AMI at the lowest and highest heart rates. Alternatively, this observation could have arisen as a result of an artefact of the data, for example if a substantial proportion of women in the low RHR group failed to report the use of rate limiting medications used in the management of underlying ischaemic heart disease. Definitive explanation of this observation requires dedicated study and is beyond the scope of the current work. Importantly, for both men and women, the positive RHR-all-cause mortality association was not fully explained by excess CVD events. Other studies have identified non-cardiac conditions as important components of excess mortality related to elevated RHR [10], with cancer mortality being a dominant cause [11,27]. Consistent with this, we observed 18% and 15% increased hazard of cancer mortality with every 10bpm increment of RHR in men and women respectively.

Several smaller studies have explored the modifying effect of age on the relationship between RHR and mortality and CVD outcomes with discrepant results [10,13,16]. There are limited reports of a similar effect size of RHR for all-cause mortality in different age groups [10], one report of a reverse relationship [16], and several studies suggesting larger effect sizes at younger ages [17,18]. Our findings, in a much larger cohort, support this latter interaction. However, interestingly, this age-RHR interaction was much less clear for CVD, IHD or AMI mortality, with the effect size greatest in the middle age band, rather than a linear decrease in effect size with age. Given the convincing interaction between RHR and age on incident AMI, which appeared to be of greater relative magnitude than those for all-cause mortality or cancer mortality, we hypothesise that age may influence the relationship between RHR and the occurrence of AMI, but once an AMI has occurred, the subsequent risk of death is less dependent on, or differently influenced by, prior RHR. The former suggestion seems an intuitively reasonable supposition from the clinical point of view, but will of course require further exploration, as will the possibility of a differential interaction between RHR and age on incident AMI vs fatal AMI.

### Possible aetiological pathways for the association of RHR and CVD

The scientific literature on mechanisms by which RHR may increase CVD mortality is limited, however several potential explanations may be considered (Fig 1). Firstly, a faster RHR is likely to have adverse haemodynamic consequences leading to increased vascular shear stress, myocardial mechanical load, and tensile stress [28,29]. Together, these haemodynamic alterations promote adverse vascular and myocardial remodelling, thereby increasing the likelihood of endothelial injury, development of atherosclerotic disease, and heart failure syndromes. There are several possibilities regarding the underlying cause of a faster RHR. Some individuals may have an intrinsically faster RHR due to differences in autonomic nervous system balance, specifically, increased sympathetic tone [30]. In such cases, faster RHR would act as the proponent of increased cardiovascular risk mediated by the associated adverse haemodynamic effects. Alternatively, elevated RHR may be downstream of some underlying biological process. There are multitudes of possible driving processes, such as, inflammatory cascades, corticosteroid levels, and sex hormone levels. These biological processes may have mechanistic roles in the genesis of CVD, perhaps initiating endothelial injury and atherogenesis or causing direct cardiotoxicity [30–32]. Alternatively, the effect of these systemic processes on CVD may be indirect, mediated solely through the adverse haemodynamic impact of an elevated RHR. It is, of course, also feasible that the elevations in RHR are representative of early subclinical CVD, and the observed relationship with CVD mortality is due to reverse causation.

Our data supports differential causative disease pathways in men and women, with greater effect from ischaemic cardiac disease in men and non-ischaemic disease in women. With regards the increased hazard of cancer mortality, reverse causation seems a likely explanation. However, it is also possible that elevated RHR is an indicator of biological pathways that predispose or are precursors to subsequent development of cancer.

## Strengths and limitations

The strengths of this study include its large sample size, detailed characterisation of participants, standardised RHR measurement, and validated prospective outcome data. However, as this is an observational study, we cannot exclude the possibility of residual confounding or reverse causation. We consider heart rate measured at a single time point at the UKB baseline assessment visit. Out-of-office measurements of heart rate may provide a more accurate representation of an individual’s resting heart rate and thus a better measure of the exposure. This approach has not yet been addressed by existing research, presumably due to limitations in obtaining a standardised out-of-office measure of heart rate, and cost and feasibility issues relating to recruitment of a large sample with long follow up. Such studies may become feasible with the increased use of personal monitoring devices such as the Apple Watch^®^ and certainly warrant consideration as an area of future research. The very large sample in our study is likely to mitigate small variations in the data and although there may be some inaccuracies relating to measurement of the exposure, this is extremely unlikely to have influenced the direction of observed effects or the overall conclusions of the study. Our cohort had a restricted age range and we cannot exclude the possibility of no relationship at very old, or very young age. Some of the covariates, such as medication use, are based on self-report and therefore may be subject to bias. young age. In addition, we cannot comment on ethnic variations, as the UKB cohort comprises a predominantly Caucasian population.

## Conclusions

In this prospective study of 228,594 men and 272,737 women with 7–12 years follow-up and verified outcomes, we confirm RHR as an independent predictor of all-cause and CVD mortality, with differential associations by age, sex, and disease. Effect sizes for all-cause mortality, cancer mortality, and incident AMI were greater at younger ages. In men, ischaemic cardiac outcomes were important drivers of the increased all-cause mortality. In women, non-ischaemic outcomes were more significant. Our findings, in the largest cohort studied internationally to date, support the consideration of RHR assessment in risk stratification algorithms, and inform our understanding of potential underlying mechanisms in the pathoaetiology of cardiovascular disease.

## Supporting information

S1 Table Full list of rate-modifying medications identified from self to report and included in the fully adjusted model. (DOCX)

S2 Table Baseline participant characteristics by quartile of resting heart rate. (DOCX)

S3 Table Cox proportional hazard models and sub-distribution hazard models for all resting heart rate-outcome relationships (systolic blood pressure as covariate). ^**†**^Results are hazard ratio (95% confidence interval) for all-cause mortality and sub-distribution hazard ratio (95% confidence interval) for all other outcomes per 10 beat per minute increase in resting heart rate. Significance level is p-value <0.0008. Those with prevalent MI have been excluded from analysis of incident AMI and fatal AMI. * CVD risk factors include: diabetes, systolic blood pressure, hypercholesterolaemia, smoking, BMI, Townsend deprivation index. **rate modifying medications include: betablockers, non-dihydropyridine calcium channel blockers, oral nitrates, digoxin, flecainide, amiodarone. AMI: acute myocardial infarction; BMI: body mass index; CVD: cardiovascular disease; IHD: ischaemic heart disease.
(DOCX)

S4 Table Testing for non-linearity of RHR effect on all outcomes in men and women. *Results are from the fully adjusted model, including following covariates: age, diabetes, hypertension, hypercholesterolaemia, smoking, BMI, Townsend deprivation index, and rate modifying medications (betablockers, non-dihydropyridine calcium channel blockers, oral nitrates, digoxin, flecainide, amiodarone). AMI: acute myocardial infarction; CVD: cardiovascular disease; IHD: ischaemic heart disease.
(DOCX)
