# Supplementary material for: Age, sex and disease-specific associations between resting heart rate and cardiovascular mortality in the UK BIOBANK
Source: PLoS One. 2020 May 29;15(5):e0233898. doi: 10.1371/journal.pone.0233898 (PMC7259773; doi:10.1371/journal.pone.0233898)
Supplement: S1 Table — (DOCX) [file pone.0233898.s001.docx]

**S1 Table. Full list of rate-modifying medications identified from self to report and included in the fully adjusted model.**

| Oral beta to blocker preparations |
| --- |
| Atenolol |
| bisoprolol |
| metoprolol |
| carvedilol |
| propranolol |
| inderal 10mg tablet |
| apsolol 10mg tablet |
| propanix 10mg tablet |
| sotalol |
| nebivolol |
| dorzolamide+timolol |
| atenolol+bendroflumethiazide |
| latanoprost+timolol |
| betaxolol |
| atenolol+bendrofluazide |
| nadolol |
| prindolol |
| Timolol |
| ethambutolol |
| pindolol |
| atenolol+chlortalidone |
| atenolol+nifedipine 50mg/20mg m/r capsule |
| atenolol+chlorthalidone |
| atenolol+co to amilozide |
| nadolol+bendroflumethiazide 40mg/5mg tablet |
| timolol maleate+bendroflumethiazide 10mg/2.5mg tablet |
| bisoprolol fumarate+hydrochlorothiazide 10mg/6.25mg tablet |
| celiprolol |
| labetalol |
| oxprenolol |
| acebutolol |
| propranolol hydrochloride+bendrofluazide 80mg/2.5mg capsule |
| sotalol hydrochloride+hydrochlorothiazide 80mg/12.5mg tablet |
| metoprolol tartrate+hydrochlorothiazide 100mg/12.5mg tablet |
| beta to blocker |
| tenormin 25 tablet |
| bedranol 10mg tablet |
| levobunolol |
| sotalol hydrochloride+hydrochlorothiazide 80mg/12.5mg tablet |
| propranolol hydrochloride+bendrofluazide 80mg/2.5mg capsule |
| carteolol |
| metoprolol tartrate+chlorthalidone 100mg/12.5mg tablet |
| half beta to prograne 80mg m/r capsule |
| beta to prograne 160mg m/r capsule |
| timolol maleate+co to amilozide 10mg/2.5mg/25mg tablet |
| cardinol 10mg tablet |
| Non to dihydropyridine calcium channel blocker preparations |
| cordilox 40mg tablet |
| adizem to xl plus m/r capsule |
| diltiazem |
| tildiem 60mg m/r tablet |
| verapamil |
| dilzem sr 60mg long acting m/r capsule |
| diltiazem hcl+hydrochlorothiazide 150mg/12.5mg m/r capsule |
| adizem to 60 m/r tablet |
| slozem 120mg m/r capsule |
| viazem xl 120mg m/r capsule |
| zemtard 120 xl m/r capsule |
| metazem 60mg m/r tablet |
| bi to carzem sr 60mg m/r capsule |
| adizem to xl plus m/r capsule |
| Angitil sr 90 m/r capsule |
| Oral nitrate preparations (excluding GTN spray/sl) |
| mycardol 30mg tablet |
| elantan 10 tablet |
| isosorbide mononitrate |
| ismn to isosorbide mononitrate |
| imdur 60mg durule |
| half to inderal la 80mg m/r capsule |
| isosorbide dinitrate |
| ismo to isosorbide mononitrate |
| isosorbide mononitrate product |
| xismox xl 60 m/r tablet |
| monomil xl 60mg m/r tablet |
| monomax sr 40 m/r capsule |
| isib 20mg tablet |
| ismo 10 tablet |
| isdn to isosorbide dinitrate |
| Other rate modifying drugs |
| amiodarone |
| digoxin |
| flecainide |
| digoxin product |
| medigoxin |
