# Supplementary material for: Age, sex and disease-specific associations between resting heart rate and cardiovascular mortality in the UK BIOBANK
Source: PLoS One. 2020 May 29;15(5):e0233898. doi: 10.1371/journal.pone.0233898 (PMC7259773; doi:10.1371/journal.pone.0233898)
Supplement: S2 Table — (DOCX) [file pone.0233898.s002.docx]

**S2 Table. Baseline participant characteristics by quartile of resting heart rate.**

|  | Quartile of resting heart rate | Whole cohort  *n*= 502,534 | Men  *n*=228,594 | Women  *n*=272,737 |
| --- | --- | --- | --- | --- |
| Age (years) | 1  2  3  4 | 56.7 (8.1)  56.4 (8.0)  56.3 (8.1)  56.7 (8.1) | 57.0 (8.3)  56.7 (8.2)  56.5 (8.2)  56.7 (8.1) | 56.3 (7.9)  56.2 (7.9)  56.2 (8.1)  56.7 (8.1) |
| Townsend deprivation index | 1  2  3  4 | -1.51 (2.97)  -1.41 (3.03)  -1.26 (3.11)  -1.00 (3.24) | -1.58 (4.66)  -1.39 (2.00)  -1.17 (2.15)  -0.75 (8.01) | -1.43 (2.99)  -1.42 (2.99)  -1.32 (2.14)  -1.18 (7.29) |
| Current smoker | 1  2  3  4 | 10218 (8.1%)  12148 (9.4%)  13605 (11.0%)  16861 (13.9%) | 5080 (8.7%)  6162 (11.0%)  7869 (13.4%)  9433 (17.2%) | 5062 (7.2%)  5544 (7.9%)  6175 (9.4%)  7507 (11.4%) |
| Body mass index ( kg/m^2^) | 1  2  3  4 | 26.7 (4.2)  27.0 (4.4)  27.5 (4.8)  28.6 (5.4) | 27.1 (3.8)  27.6 (4.0)  28.1 (4.3)  28.9 (4.8) | 26.2 (4.6)  26.5 (4.7)  27.2 (5.1)  28.4 (5.8) |
| Systolic blood pressure (mmHg) | 1  2  3  4 | 136.7 (18.6)  136.5 (18.4)  137.5 (18.4)  140.9 (18.9) | 139.0 (17.6)  140.2 (17.0)  141.4 (17.0)  143.8 (17.9) | 133.8 (19.4)  133.7 (19.0)  134.9 (18.9)  138.7 (19.2) |
| Diastolic blood pressure (mmHg) | 1  2  3  4 | 79.3 (9.5)  81.2 (9.7)  82.9 (9.9)  85.8 (10.3) | 80.9 (9.4)  83.6 (9.6)  85.3 (9.7)  87.7 (10.1) | 77.2 (9.3)  79.4 (9.4)  81.2 (9.7)  84.4 (10.1) |
| Diabetes | 1  2  3  4 | 4684 (3.7%)  5005 (3.9%)  6247 (5.1%)  10793 (8.8%) | 2716 (4.7%)  2992 (5.3%)  4195 (7.1%)  6320 (11.5%) | 1766 (2.5%)  1791 (2.6%)  2378 (3.6%)  4571 (6.9%) |
| Hypertension | 1  2  3  4 | 34538 (27.4%)  31237 (24.1%)  32717 (26.5%)  42271 (34.6%) | 17828 (30.6%)  15955 (28.3%)  18779 (31.8%)  22148 (40.3%) | 16425 (23.3%)  14369 (20.5%  15065 (22.8%)  20194 (30.5%) |
| Hypercholesterolaemia | 1  2  3  4 | 25529 (20.3%)  21039 (16.2%)  21094 (17.1%)  25998 (21.3%) | 14924 (25.6%)  12245 (21.7%)  13516 (22.9%)  14961 (27.2%) | 9775 (13.9%)  8431 (12.0%)  8642 (13.1%)  11166 (16.9%) |
| Resting heart rate (bpm) | 1  2  3  4 | 56.2 (4.4)  65.3 (2.0)  72.3 (2.1)  84.6 (7.6) | 55.7 (4.7)  65.2 (2.0)  72.2 (2.1)  85.1 (8.0) | 56.9 (4.0)  65.4 (2.0)  72.3 (2.1)  84.2 (7.3) |
| Physical Activity (metabolic equivalent minutes/week) | 1  2  3  4 | 2142 [1053-4151]  1866 [874-3732]  1691 [762-3453]  1493 [654-3172] | 2253 [1116-4332]  1935 [890-3930]  1716 [753-3653]  1466 [600-3255] | 2044 [998-3906]  1830 [864-3547]  1662 [773-3306]  1510 [690-3093] |
| Follow-up (days):  Incident AMI | 1  2  3  4 | 8.9 [8.2-9.6]  8.9 [8.2-9.6]  9.0 [8.3-9.7]  9.0 [8.3-9.7] | 9.0 [8.2-9.6]  8.9 [8.2-9.6]  9.0 [8.2-9.7]  9.0 [8.2-9.7] | 9.0 [8.2-9.6]  9.0 [8.3-9.7]  9.0 [8.3-9.7]  9.0 [8.3-9.7] |
| Follow-up (days):  Incident AMI | 1  2  3  4 | 8.1 [7.4-8.8]  8.1 [7.4-8.8]  8.2 [7.5-8.9]  8.2 [7.5-8.9] | 8.1 [7.4-8.8]  8.1 [7.4-8.8]  8.2 [7.4-8.9]  8.2 [7.5-8.9] | 8.1 [7.4-8.8]  8.1 [7.5-8.8]  8.2 [7.5-8.9]  8.3 [7.5-8.9] |
