# Supplementary material for: Age, sex and disease-specific associations between resting heart rate and cardiovascular mortality in the UK BIOBANK
Source: PLoS One. 2020 May 29;15(5):e0233898. doi: 10.1371/journal.pone.0233898 (PMC7259773; doi:10.1371/journal.pone.0233898)
Supplement: S3 Table — †Results are hazard ratio (95% confidence interval) for all-cause mortality and sub-distribution hazard ratio (95% confidence interval) for all other outcomes per 10 beat per minute increase in resting heart rate. Significance level is p-value <0.0008. Those with prevalent MI have been excluded from analysis of incident AMI and fatal AMI. * CVD risk factors include: diabetes, systolic blood pressure, hypercholesterolaemia, smoking, BMI, Townsend deprivation index. **rate modifying medications include: betablockers, non-dihydropyridine calcium channel blockers, oral nitrates, digoxin, flecainide, amiodarone. AMI: acute myocardial infarction; BMI: body mass index; CVD: cardiovascular disease; IHD: ischaemic heart disease. (DOCX) [file pone.0233898.s003.docx]

**S3 Table. Cox proportional hazard models and sub-distribution hazard models for all resting heart rate-outcome relationships (systolic blood pressure as covariate)^†^ .**

|  | Model 4: Model 3 + CVD risk factors^*^ | Model 5: Model 4 + rate modifying medications^**^ |
| --- | --- | --- |
| All-cause mortality |  |  |
| Men | 1.19 (1.17 to 1.21) | 1.22 (1.20 to 1.24) |
| p-value | 1.4 $\times$10^-93^ | 1.1 $\times$10^-124^ |
| Women | 1.15 (1.13 to 1.18) | 1.19 (1.16 to 1.22) |
| p-value | 5.8 $\times$10^-31^ | 1.2 $\times$10^-45^ |
| CVD mortality |  |  |
| Men | 1.10 (1.07 to 1.14) | 1.18 (1.14 to 1.22) |
| p-value | 6.3 $\times$10^-9^ | 1.2 $\times$10^-21^ |
| Women | 1.07 (1.00 to 1.14) | 1.16 (1.08 to 1.23) |
| p-value | 0.047 | 0.00001 |
| IHD mortality |  |  |
| Men | 1.06 (1.01 to 1.11) | 1.15 (1.10 to 1.20) |
| p-value | 0.009 | 1.7 $\times$10^-10^ |
| Women | 0.97 (0.87 to 1.09) | 1.07 (0.96 to 1.19) |
| p-value | 0.59 | 0.25 |
| Fatal AMI |  |  |
| Men | 1.04 (0.97 to 1.12) | 1.13 (1.05 to 1.21) |
| p-value | 0.25 | 0.0008 |
| Women | 0.84 (0.70 to 1.01) | 0.92 (0.77 to 1.10) |
| p-value | 0.059 | 0.36 |
| Incident AMI |  |  |
| Men | 1.01 (0.98 to 1.03) | 1.05 (1.02 to 1.07) |
| p-value | 0.50 | 0.0002 |
| Women | 0.98 (0.94 to 1.03) | 1.02 (0.98 to 1.07) |
| p-value | 0.40 | 0.29 |
| Cancer mortality |  |  |
| Men | 1.17 (1.15 to 1.20) | 1.19 (1.16 to 1.21) |
| p-value | 3.2 $\times$10^-42^ | 5.6 $\times$10^-46^ |
| Women | 1.13 (1.10 to 1.17) | 1.15 (1.11 to 1.18) |
| p-value | 1.8 $\times$10^-16^ | 9.6 $\times$10^-19^ |

**^†^**Results are hazard ratio (95% confidence interval) for all-cause mortality and sub-distribution hazard ratio (95% confidence interval) for all other outcomes per 10 beat per minute increase in resting heart rate. Significance level is p-value <0.0008. Those with prevalent MI have been excluded from analysis of incident AMI and fatal AMI. ^*^ CVD risk factors include: diabetes, systolic blood pressure, hypercholesterolaemia, smoking, BMI, Townsend deprivation index. ^**^rate modifying medications include: betablockers, non-dihydropyridine calcium channel blockers, oral nitrates, digoxin, flecainide, amiodarone. AMI: acute myocardial infarction; BMI: body mass index; CVD: cardiovascular disease; IHD: ischaemic heart disease
