# Supplementary material for: Age, sex and disease-specific associations between resting heart rate and cardiovascular mortality in the UK BIOBANK
Source: PLoS One. 2020 May 29;15(5):e0233898. doi: 10.1371/journal.pone.0233898 (PMC7259773; doi:10.1371/journal.pone.0233898)
Supplement: S4 Table — *Results are from the fully adjusted model, including following covariates: age, diabetes, hypertension, hypercholesterolaemia, smoking, BMI, Townsend deprivation index, and rate modifying medications (betablockers, non-dihydropyridine calcium channel blockers, oral nitrates, digoxin, flecainide, amiodarone). AMI: acute myocardial infarction; CVD: cardiovascular disease; IHD: ischaemic heart disease. (DOCX) [file pone.0233898.s004.docx]

**S4 Table. Testing for non-linearity of RHR effect on all outcomes in men and women.**

|  | p-value non-linear vs. linear model for RHR^*^ |
| --- | --- |
| All-cause mortality | 0.39 |
| Men | 0.94 |
| Women | 0.13 |
| CVD mortality | 0.11 |
| Men | 0.21 |
| Women | 0.38 |
| IHD mortality | 0.06 |
| Men | 0.14 |
| Women | 0.21 |
| AMI mortality | 0.78 |
| Men | 0.69 |
| Women | 0.66 |
| Incident AMI | **0.03** |
| Men | 0.53 |
| Women | **0.002** |
| Cancer mortality | 0.59 |
| Men | 0.32 |
| Women | 0.41 |

^*^Results are from the fully adjusted model, including following covariates: age, diabetes, hypertension, hypercholesterolaemia, smoking, BMI, Townsend deprivation index, and rate modifying medications (betablockers, non-dihydropyridine calcium channel blockers, oral nitrates, digoxin, flecainide, amiodarone). AMI: acute myocardial infarction; CVD: cardiovascular disease; IHD: ischaemic heart disease
